# Temperature-Related Yield Constraints of Early-Rice in South China: A Cross-Location Analysis

**DOI:** 10.1371/journal.pone.0158601

**Published:** 2016-07-01

**Authors:** Min Huang, Ruichun Zhang, Peng Jiang, Xiaobing Xie, Xuefeng Zhou, Fangbo Cao, Yingbin Zou

**Affiliations:** 1 Southern Regional Collaborative Innovation Center for Grain and Oil Crops (CICGO), Hunan Agricultural University, Changsha, China; 2 Hengyang Institute of Agricultural Sciences, Henyang, China; 3 Rice and Sorghum Research Institute, Sichuan Academy of Agricultural Sciences, Deyang, China; 4 Key Laboratory of Southwest Rice Biology and Genetic Breeding, Ministry of Agriculture, Deyang, China; Louisiana State University Agricultural Center, UNITED STATES

## Abstract

Warm temperature during post-heading is generally hypothesized to be the critical factor limiting grain yield of early-rice in South China. However, there is no direct evidence to confirm this hypothesis in the field. This study was conducted to determine the temperature-related yield constraints of early-rice in South China. Field experiments were carried out in Huaiji (a location in South China) and Changsha (a location in the Yangtze River basin) in 2011–2013. In each year, two rice cultivars were grown in early-rice growing season in Huaiji and in single-rice growing season in Changsha. Huaiji had higher average daily maximum temperature during post-heading than Changsha. The higher temperature during post-heading induced early plant senescence (slower crop growth rate and shorter grain filling duration), but grain weight did not reduce because it was compensated for by increased translocation of pre-heading biomass. The higher temperature during post-heading also did not cause a reduction in grain filling percentage. Huaiji had lower temperature during pre-heading than Changsha, which to some extent resulted in slower crop growth rate and consequently lower biomass production and smaller sink size in Huaiji than in Changsha. As a result, grain yield was about 30% lower in Huaiji than in Changsha. Our results indicate that grain yield of early-rice in South China is limited not by warm temperature during post-heading but partially by cool temperature during pre-heading, and suggest that enhancing sink size and meanwhile maintaining good translocation of pre-heading biomass may be an effective way to achieve high yield for early-rice in South China.

## Introduction

Rice is the main staple crop for about 65% of the population in China. It is widely grown in South China, the Yangtze River basin and Northeast China [1]. Due to the differences in climate conditions, grain yields of rice highly vary depending on regions [2, 3]. There is view that climate conditions in South China are not favorable for achieving high grain yield of rice [4]. Typically, in the early-rice growing season (from March to July), warm temperature during post-heading is hypothesized to be the critical factor limiting grain yield. However, there is no direct evidence to confirm this hypothesis in the field.

There have been reports regarding the effects of warm temperature during post-heading on growth and yield attributes of rice. In general, warm temperature during post-heading hastens plant senescence, shortens grain filling duration, and reduces biomass production and grain weight [5, 6, 7]. However, grain filling depends not only on the newly assimilated biomass during post-heading but also on the biomass translocated from pre-heading stored reserves [8, 9]. It has been reported that the negative effect of warm temperature on grain filling can be overcame by better biomass translocation [10, 11]. In addition, Liu et al. [12] observed that warm temperature during post-heading also caused a significant reduction in grain filling percentage. In this regard, it is well documented that warm temperature can result in increased pollen sterility and consequently decreased spikelet fertility [13, 14].

Cross-location analysis is a useful approach for determining the climatic factors and physiological processes that limit grain yield [15, 16]. There are many rice cultivars bred in South China that have been widely grown in the single-rice growing season (from May to September) in the Yangtze River basin, and these cultivars generally produce higher grain yields in the Yangtze River basin than in South China. In the present study, growth and yield attributes of two rice cultivars bred in South China were compared between two locations, one in South China and one in the Yangtze River basin. Our objective was to determine the temperature-related yield constraints of early-rice in South China.

## Material and Methods

### Ethics statements

No specific permissions were required for the activities conducted in this study. The field used in this study is neither privately owned nor protected. The experiments did not involve endangered or protected species.

### Experimental details

Field experiments were conducted in early-rice growing season in Huaiji (24°04′ N, 112°03′ E) and in single-rice growing season in Changsha (28°11′ N, 113°04′ E), China in 2011–2013. Huaiji and Changsha are located in South China and the Yangtze River basin, respectively. The soils in the two locations were Ultisols (USDA taxonomy) [17]. The soil properties in Huaiji were: pH = 5.24, organic matter = 33.8 g kg^–1^, total N = 1.68 g kg^–1^, available P = 9.16 mg kg^–1^, and available K = 77.2 mg kg^–1^; and in Changsha were: pH = 5.83, organic matter = 27.7 g kg^–1^, total N = 1.59 g kg^–1^, available P = 54.5 mg kg^–1^, and available K = 63.2 mg kg^–1^. The soil test was based on samples taken from the upper 20 cm of the soil.

Two rice cultivars, Huanghuazhan and Yuxiangyouzhan, were used in this study. Both Huanghuazhan and Yuxiangyouzhan were bred by Guangdong Academy of Agricultural Sciences located in South China. Huanghuazhan was developed from a cross of Huangxinzhan/Fenghuazhan, while Yuxiangyouzhan from a backcross of TY36/IR100//IR100. These two cultivars have been widely grown by farmers in South China and the Yangtze River basin. Treatments were arranged in a split plot design with two N treatments as main plots and two rice cultivars as subplots. The experiment was replicated three times and subplot size was 20 m^2^. Two N treatments were high and moderate N rates. In the high N rate, a total N rate of 225 kg ha^–1^ was applied. In the moderate N rate, a total N rate of 112.5 kg ha^–1^ was applied in 2011, while in 2012 and 2013 a chlorophyll meter (SPAD-502, Minolta Camera Co., Tokyo, Japan) was used to determine leaf N status for making decisions on topdressed N application. Detailed information about N application timing and rate was given in Table 1.

**Table 1:** Information about two N treatments in Huaiji and Changsha in 2011–2013.

N treatment	Year	N application timing and rate (kg ha^–1^)^a^				Total N rate(kg ha^–1^)
		Basal	Early tillering	Panicle initiation	Spikelet differentiation	
High N	2011–2013	112.5^HC^	45^HC^	45^HC^	22.5^HC^	225^HC^
Moderate N^b^	2011	56.25^HC^	22.5^HC^	22.5^HC^	11.25^HC^	112.5^HC^
	2012	56^HC^	60^HC^	30^H^/45^C^	15^H^/0^C^	161^H^/161^C^
	2013	56^HC^	60^HC^	60^H^/45^C^	15^H^/0^C^	191^H^/161^C^

^HC^ denotes Huaiji and Changsha; ^H^ denotes Huaiji; ^C^ denotes Changsha.

^a^Basal was defined as 1 day before transplanting, early tillering as 7 days after transplanting, panicle initiation as the first appearance of differentiated apex), and spikelet differentiation as the appearance of glumous flower primordial at the tips of elongating primary rachis-branches.

^b^In 2012 and 2013, N rates at panicle initiate and spikelet differentiation was based on chlorophyll meter reading (SPAD) [26]. SPAD was measured on 10 topmost fully expanded leaves in each subplot. At panicle initiation, if SPAD was below 37, 60 kg ha^–1^ was applied; if SPAD was between 37 and 39, 45 kg ha^–1^ was applied; if SPAD was above 39, 30 kg ha^–1^ was applied. At spikelet differentiation, if SPAD was below 37, 45 kg ha^–1^ was applied; if SPAD was between 37 and 39, 30 kg ha^–1^ was applied; if SPAD was between 39 and 42, 15 kg ha^–1^ was applied; if SPAD was above 42, 0 kg ha^–1^ was applied.

Pre-germinated seeds were sown in a seedbed. Field preparation was carried out by two rotary plowing operations. Seedlings with 4–5 leaves were transplanted at a spacing of 27 cm × 20 cm, with two seedlings per hill. Phosphorus (112.5 kg P_2_O_5_ ha^–1^) were applied and incorporated in all subplots 1 day before transplanting. Potassium (157.5 kg K_2_O ha^–1^) was split equally at basal and panicle initiation. The regime for water management was in the sequence of shallow irrigation (2–3 cm), midseason drainage (10–15 d) and shallow irrigation. Diseases and insects were intensively controlled by chemicals. Weeds were controlled by a combination of herbicide and hand-weeding. No heavy infestations were recorded in the experiments. Dates of seed sowing, transplanting, heading and maturity were provided in Table 2.

**Table 2:** Dates of seed sowing, transplanting, heading and maturity in Huaiji and Changsha in 2011–2013.

Year	Location	Seed sowing date	Transplanting date	Heading date	Maturity date
2011	Huaiji	10 March	8 April	20 June	14 July
	Changsha	13-May	5 June	7 August	11 September
2012	Huaiji	10 March	11 April	22 June	16 July
	Changsha	13 May	12 June	14 August	17 September
2013	Huaiji	10 March	5 April	17 June	13 July
	Changsha	13 May	8 June	12 August	16 September

Six hills were sampled in each subplot at heading. Plants were oven-dried at 70°C to constant weight to determine pre-heading biomass production. Ten hills were diagonally sampled from a 5-m^2^ harvest area for each subplot at maturity to determine yield components (panicles m^–2^, spikelets panicle^–1^, spikelets m^–2^, grain filling percentage and grain weight), biomass production, and harvest index. Panicle number was counted in each hill. Plants were separated into straw (including rachis) and spikelets by hand threshing. Filled spikelets were separated from unfilled spikelets by submerging them in tap water [18]. Three subsamples of 30 g of spikelets and all unfilled spikelets were taken to count the number of spikelelts. Dry weights of straw, and filled and unfilled spikelets were determined after over-drying at 70°C to constant weight. Total biomass production was the total dry matter of straw, and filled and unfilled spikelets. Post-heading biomass production was the difference between total biomass production and pre-heading biomass production. Pre-heading crop growth rate (CGR), post-heading CGR, source-sink ratio at heading, amount of translocated biomass in grain (A_T_), amount of post-heading biomass in grain (A_P_) and grain filling rate were calculated according to following formulas:

- Pre-heading CGR = Pre-heading biomass production/Growth duration from transplanting to heading
- Post-heading CGR = Post-heading biomass production/Growth duration from heading to maturity
- Source-sink ratio at heading = Pre-heading biomass production/Spikelets m^–2^
- A_T_ = (Filled spikelet weight–Post-heading biomass production)/Filled spikelets m^–2^
- A_P_ = Post-heading biomass production/Filled spikelets m^–2^
- Grain filling rate = Grain weight/Growth duration from heading to maturity

Grain yield was determined from a 5-m^2^ area in each subplot and adjusted to the standard moisture content of 0.14 g H_2_O g^–1^. Daily maximum and minimum temperatures were taken from the local meteorological stations. Daily effective temperature was calculated by subtracting a base temperature (10°C) from the average of the daily maximum and minimum temperatures [19]. Total effective temperature was calculated as the sum of the daily effective temperature.

### Statistical analysis

Data were analyzed following analysis of variance (Statistix 8.0 software package, Tallahassee, Florida, USA). The statistical model included the main effect of location, N treatment, cultivar, and their interactions. Means were compared based on the least significant difference test (LSD) at the 0.05 probability level.

## Results

**Fig 1:**
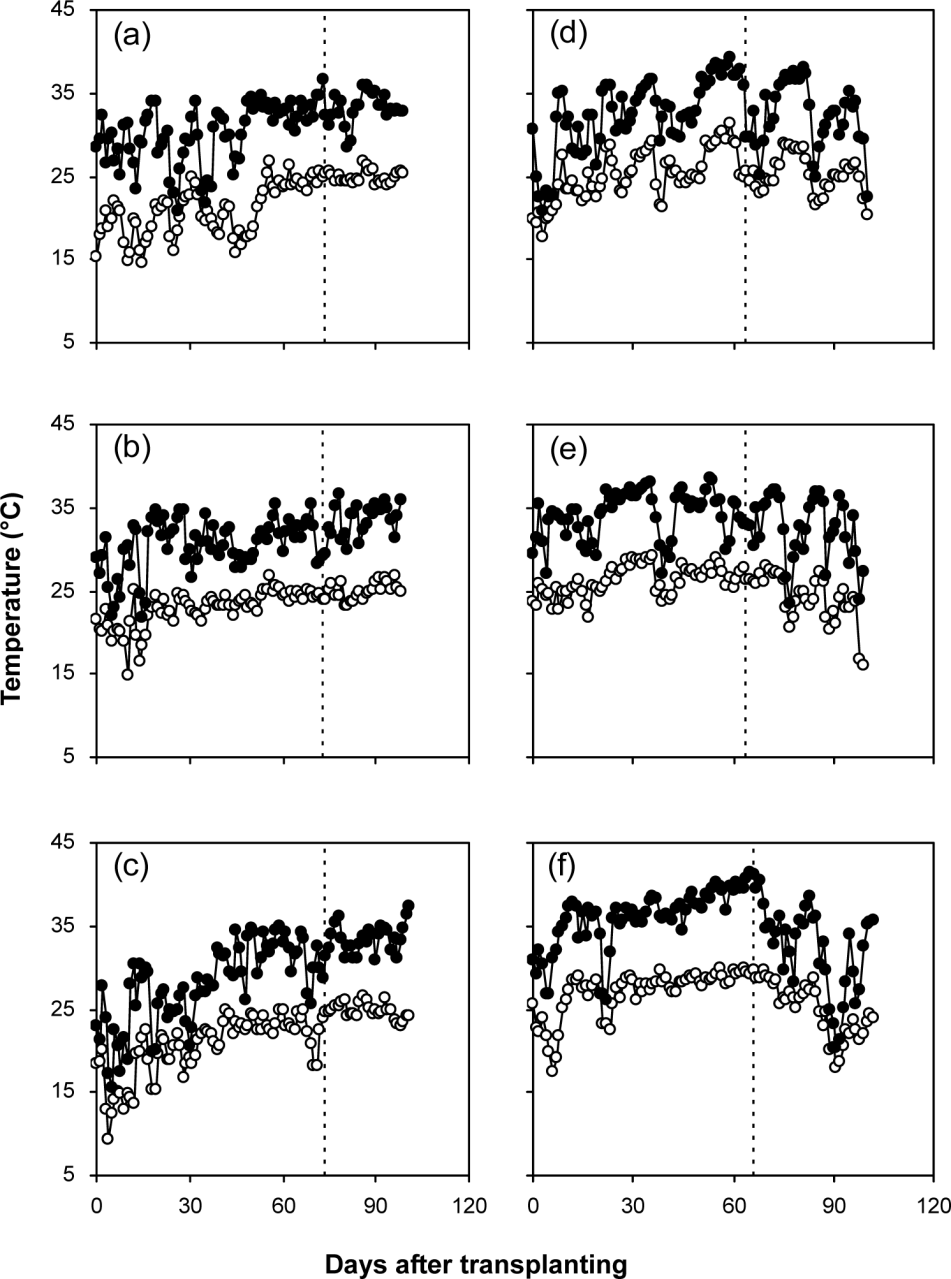
**Daily maximum (close circles) and minimum temperatures (open circles) during early-rice growing season in Huaiji (a–c) and during single-rice growing season in Changsha (d–f) in 2011 (a, d), 2012 (b, e) and 2013 (c, f).** In each subfigure, vertical dot line denotes heading date.

**Fig 2:**
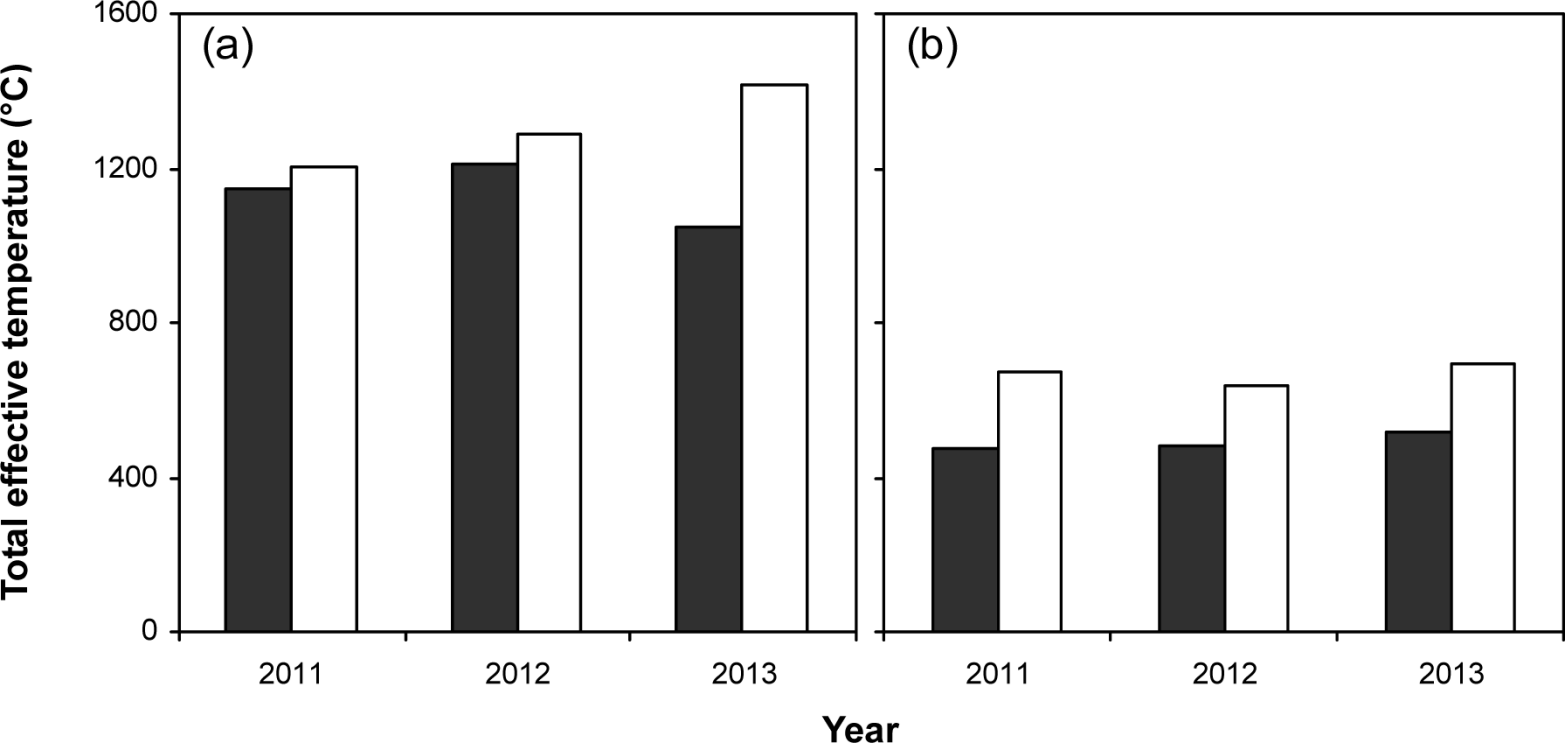
**Total effective temperature during pre-heading (a) and post-heading (b) in Huaiji (solid columns) and Changsha (open columns) in 2011–2013**.

Average daily maximum temperatures during pre-heading were lower in Huaiji (28.0–30.4°C) than in Changsha (32.3–36.0°C), whereas those during post-heading were higher in Huaiji (33.1–33.5°C) than in Changsha (32.2–32.4°C) (Fig 1A–1F). Average daily minimum temperatures during pre-heading in Huaiji (20.5–22.9°C) were lower than those in Changsha (25.1–27.0°C). The differences in average daily minimum temperatures during post-heading between the two locations were inconsistent. Huaiji had 8–10 d longer duration of pre-heading but 9–11 d shorter duration of post-heading than Changsha. Total effective temperatures were lower in Huaiji than in Changsha by 5–26% during pre-heading and by 25–29% during post-heading (Fig 2A and 2B).

The difference in grain yield between locations was significant in all three years (Table 3). Huaiji produced 25–32% lower grain yield than Changsha. A significant difference in grain yield between N rates was observed in 2011 but not in 2012 and 2013. In 2011, high N rate had 8% higher grain yield than moderate N rate. The difference in grain yield between cultivars was not significant in all three years. There were no significant interactive effects of location, N rate and cultivar on grain yield. Hence, mean data across two N rates and two cultivars were presented in subsequent tables.

**Table 3:** Grain yield (t ha^–1^) of two rice cultivars grown under two N treatments in Huaiji and Changsha in 2011–2013.

Location	Cultivar	2011		2012		2013	
		High N	Moderate N	High N	Moderate N	High N	Moderate N
Huaiji	Huanghuazhan	6.45	5.84	6.96	7.05	6.89	7.12
	Yuxiangyouzhan	7.07	6.41	6.79	6.77	7.46	6.58
	Mean	6.76	6.12	6.87	6.91	7.17	6.85
Changsha	Huanghuazhan	9.82	9.02	9.09	9.44	10.53	10.46
	Yuxiangyouzhan	9.69	9.38	8.84	9.49	9.17	9.46
	Mean	9.75	9.20	8.96	9.46	9.85	9.96
Analysis of variance							
Location (L)		**		**		**	
N rate (N)		**		NS		NS	
Cultivar (C)		NS		NS		NS	
L × N		NS		NS		NS	
L × C		NS		NS		NS	
N × C		NS		NS		NS	
L × N × C		NS		NS		NS	

** indicates significance at the 0.01 probability level.

NS indicates non-significance at the 0.05 probability level.

Panicles m^–2^ was less in Huaiji than in Changsha by 17–33% (Table 4). Spikelets panicle^–1^ was less in Huaiji than in Changsha by 18% in 2011 and by 17% in 2012, whereas the difference was not significant in 2013. Huaiji had 26–35% lower spikelets m^–2^ than Changsha. There were no significant differences in grain filling percentage and grain weight between Huaiji and Changsha in all three years.

**Table 4:** Yield components of rice grown in Huaiji and Changsha in 2011–2013.

Year	Location	Panicles m^–2^	Spikelets panicle^–1^	Spikelets m^–2^ (×10^3^)	Grain filling (%)	Grain weight (mg)
2011	Huaiji	207*	180*	40.3*	78.6	19.7
	Changsha	250	219	54.3	76.9	19.3
2012	Huaiji	222*	168*	36.8*	80.6	19.9
	Changsha	277	202	54.7	80.9	19.7
2013	Huaiji	212*	203	42.8*	75.5	19.8
	Changsha	317	212	66.2	75.7	18.3

Data are the means across two N treatments and two cultivars.

* indicates significant difference between the two locations according to LSD (0.05).

Pre-heading biomass production was lower in Huaiji than in Changsha by 11–27% (Table 5). Post-heading biomass production in Huaiji was lower than that in Changsha by 50–59%. Total biomass production was 23–39% lower in Huaiji than in Changsha. Pre-heading CGR was slower in Huaiji than in Changsha by 23–37%. Post-heading CGR in Huaiji was 27–42% slower than that in Changsha.

**Table 5:** Biomass production and crop growth rate (CGR) of rice grown in Huaiji and Changsha in 2011–2013.

Year	Location	Biomass production (g m^–2^)			CGR (g m^–2^ d^–1^)	
		Pre-heading	Post-heading	Total	Pre-heading	Post-heading
2011	Huaiji	998*	244*	1242*	13.5*	9.8*
	Changsha	1122	486	1608	17.5	13.5
2012	Huaiji	850*	281*	1131*	11.6*	11.3*
	Changsha	1171	680	1851	18.3	19.4
2013	Huaiji	911*	338*	1249*	12.3*	12.5*
	Changsha	1199	733	1932	18.2	20.4

Data are the means across two N treatments and two cultivars.

* indicates significant difference between the two locations according to LSD (0.05).

Source-sink ratio at heading was higher in Huaiji than in Changsha by 7–20% (Table 6). A_T_ was 35–70% higher in Huaiji than in Changsha. A_P_ was lower in Huaiji than in Changsha by 18–30%. Grain filling rate in Huaiji was 43–46% higher than that in Changsha. Harvest index was lower in Huaiji than in Changsha by 6% in 2011 and by 4% in 2013, while the difference was not significant in 2012.

**Table 6:** Source-sink ratio at heading, amount of translocated biomass in grain (A_T_), amount of post-heading biomass in grain (A_P_), grain filling rate and harvest index of rice grown in Huaiji and Changsha in 2011–2013.

Year	Location	Source-sink ratio at heading (mg spikelet^–1^)	A_T_ (mg grain^–1^)	A_P_ (mg grain^–1^)	Grain filling rate (mg grain^–1^ d^–1^)	Harvest index
2011	Huaiji	24.9*	12.0*	7.8*	0.79*	0.50*
	Changsha	20.7	8.9	10.4	0.54	0.53
2012	Huaiji	23.2*	10.4*	9.5*	0.80*	0.52
	Changsha	21.6	6.1	13.6	0.56	0.51
2013	Huaiji	21.6*	9.4*	10.3*	0.73*	0.51*
	Changsha	18.1	5.6	12.6	0.51	0.53

Data are the means across two N treatments and two cultivars.

* indicates significant difference between the two locations according to LSD (0.05).

## Discussion

Grain yield was about 30% lower in early-rice in Huaiji (a location in South China) than in single-rice in Changsha (a location in the Yangtze River basin). It is generally hypothesized that warm temperature during post-heading is the critical factor limiting the grain yield of early-rice in South China [4], because it can cause reduction in grain weight by hastening plant senescence [5, 6, 7] and reduction in grain filling percentage by increasing pollen sterility [12, 13, 14]. In the present study, Huaiji had higher average daily maximum temperature during post-heading but lower post-heading CGR and shorter grain filling duration than Changsha, indicating that early plant senescence was induced by warm temperature in Huaiji as compared to Changsha. In addition, the shorter grain filling duration resulted in lower total effective temperature during post-heading in Huangji than in Changsha. However, the magnitude of reduction in grain filling duration from increase in daily maximum temperature during post-heading in Huaiji seems to be incredible. Huaiji had approximately 1°C higher average daily maximum temperature during post-heading than Changsha, which resulted in about 10 d shorter grain filling duration in Huaiji than in Changsha. This magnitude is largely higher than that reported by Huang et al. [20], who observed that growth duration decreased about 3 d for each 1°C increase in average daily maximum temperature during whole growing season. These might be attributed to that (1) the average daily maximum temperatures during post-heading in the two locations in this study (32.2–33.5°C) were largely higher than the average daily maximum temperature during whole growing season in the study of Huang et al. [20] (25.4–28.6°C), and (2) there was a large difference in fluctuation pattern of daily maximum temperature during post-heading between the two locations in this study (Fig 1A–1F). More importantly, although the early plant senescence caused reductions in post-heading biomass production and A_P_ in Huaiji, grain weight in Huaiji was not reduced because it was compensated for by increased A_T_. In this regard, it is suggested that better translocation of pre-heading biomass can accelerate grain filling rate and consequently overcome the negative effects of early plant senescence [8, 9, 10, 11]. This was also the case in the present study. Under some conditions, translocation of pre-heading biomass can be improved by increasing source and/or sink strength [8, 9]. In this study, higher source-sink ratio at heading might be partially responsible for the better translocation of pre-heading biomass in Huaiji than in Changsha. On the other hand, in the present study, the higher temperature during post-heading did not cause a reduction in grain filling percentage in Huaiji as compared to Changsha. These results suggest that temperature during post-heading was not the critical climatic factor that explains the yield gap between Huaiji and Changsha.

By analyzing of yield components, it is apparent that smaller sink size (spikelets m^–2^) was responsible for the lower grain yield in Huaiji and than in Changsha. The importance of sink size in enhancing grain yield has been reported by many investigators [2, 3, 21, 22, 23], but it remains controversial whether the sink size is determined by panicle number per unit land area or panicle size (spikelets panicle^–1^) [2, 22, 23]. In this study, the smaller sink size in Huaiji was attributed to both less panicles m^–2^ and less spikelets panicle^–1^ in 2011 and 2012, but only to less panicles m^–2^ in 2013. The different performance in 2013 might be partly attributed to that a relatively high panicles m^–2^ caused a reduction in spikelets panicle^–1^ in 2013 in Changsha (Table 4), because a strong compensation mechanism generally exists between the two yield components [22, 24]. The relatively high panicles m^–2^ in Changsha in 2013 was associated with an uncommonly warm temperature during pre-heading (Fig 1F). In another approach, sink size is also closely related to biomass production during the development phase when sink size is determined [3]. Therefore, in this study, the smaller sink size in Huaiji also could be explained by the lower pre-heading biomass production than in Changsha. Biomass production is determined by CGR and growth duration, and both of them are affected by temperature. In general, cool temperature slow CGR and prolong growth duration [22]. In addition, CGR is also associated with solar radiation [24]. However, the effect of the solar radiation on CGR could not be assessed in this study, because the solar radiation data were not available at the local weather stations. In the present study, Huaiji had lower temperature during pre-heading than Changsha, which to some extent resulted in slower pre-heading CGR and consequently lower pre-heading biomass production in Huaiji than in Changsha. Furthermore, the difference in pre-heading growth performance between the two locations also could be explained by the difference in effective temperature. Total effective temperature during pre-heading was lower in Huaiji than in Changsha. These results indicate that lower temperature during pre-heading was a critical climatic factor the smaller sink size and lower grain yield in Huaiji than in Changsha.

In addition, the results in 2011 were somewhat different form those in 2012 and 2013 as a result of the change in experimental design. In 2011, decreasing N rate from 225 kg ha^–1^ (high N rate) to 112.5 kg ha^–1^ (moderate N rate) caused a significant decrease in grain yield. The decreased grain yield in the moderate N rate in 2011 was attributed to a reduction in panicles m^–2^ (data not shown). In 2012 and 2013, N management strategy for the moderate N rate treatment was different from that in 2011 (Table 1). Firstly, N rate at early tillering was increased from 22.5 kg ha^–1^ in 2011 to 60 kg ha^–1^ in 2012 and 2013. Secondly, N rates at panicle initiation and spikelet differentiation were fixed in 2011, but based on a chlorophyll meter in 2012 and 2013. As a result, total N rate for the moderate N rate treatment was increased to 161–191 kg ha^–1^ in 2012 and 2013, and no significant differences in grain yield and yield components (data not shown) were observed between the high and the moderate N rate treatments in 2012 and 2013. These results are in agreement with those in previous studies showing that N supply and crop N requirement can be well matched by using a chlorophyll meter [25, 26, 27]. Our results also highlight that decreasing N application input in rice production should be based on knowledge-based N management practices to avoid yield loss.

## Conclusions

Grain yield of early-rice in South China is limited not by warm temperature during post-heading but to some extent by cool temperature during pre-heading, mainly because (1) early plant senescence induced by warm temperature during post-heading is compensated for by increased translocation of pre-heading biomass, and (2) cool temperature during pre-heading slows CGR and consequently decreases biomass production and reduces sink size. Our study suggests that enhancing sink size and meanwhile maintaining good translocation of pre-heading biomass may be an effective way to achieve high yield for early-rice in South China.
